# Non-verbal Enrichment in Vocabulary Learning With a Virtual Pedagogical Agent

**DOI:** 10.3389/fpsyg.2020.533839

**Published:** 2020-11-24

**Authors:** Astrid M. Rosenthal-von der Pütten, Kirsten Bergmann

**Affiliations:** ^1^Department of Society, Technology, and Human Factors, Faculty of Philosophy, RWTH Aachen University, Aachen, Germany; ^2^Faculty of Engineering and Mathematics, FH Bielefeld University of Applied Sciences, Bielefeld, Germany

**Keywords:** second language, vocabulary learning, non-verbal enrichment, word classes, pedagogical agents, gestures

## Abstract

Non-verbal enrichment in the form of pictures or gesture can support word learning in first and foreign languages. The present study seeks to compare the effects of viewing pictures vs. imitating iconic gestures on learning second language (L2) vocabulary. In our study participants learned L2 words (nouns, verbs, and adjectives) together with a virtual, pedagogical agent. The to-be-learned items were either (i) enriched with pictures, or (ii) with gestures that had to be imitated, or (iii) without any non-verbal enrichment as control. Results showed that gesture imitation was particularly supportive for learning nouns, whereas pictures showed to be most beneficial for memorizing verbs. These findings, suggesting that the type of vocabulary learning strategy has to match with the type of linguistic material to be learned, have important educational implications for L2 classrooms and technology-enhanced tutoring systems.

## 1. Introduction

The integration of pedagogical agents into technology-enhanced tutoring systems has been under research for over 20 years, but results on learning gain have often been heterogeneous (cf. Clarebout and Heidig, 2012; Schroeder et al., 2013; Johnson and Lester, 2016). Recently, however, the potential of the agent's non-verbal gestures to support the learning process has attracted notice in the domain of math education (e.g., Anasingaraju et al., 2016; Cook et al., 2017), and also for vocabulary learning (e.g., Bergmann and Macedonia, 2013). In fact, learning environments incorporating embodied agents provide a flexible way to support vocabulary learning with non-verbal enrichment—be it by gestures performed by the agent or by other means, such as pictures.

Previous research has demonstrated that picture-based enrichment (Bull and Wittrock, 1973; Smith et al., 1987, 1994; Plass et al., 1998; McGregor et al., 2007; Morett, 2019; Andrä et al., 2020) as well as gesture-based enrichment can improve word learning (with studies predominantly using iconic or emblematic gestures, cf. Quinn-Allen, 1995; Kelly et al., 2009; Macedonia and Knösche, 2011; de Nooijer et al., 2013, 2014; Macedonia and Klimesch, 2014; Baills et al., 2019; Huang et al., 2019; Andrä et al., 2020; Sweller et al., 2020). The details of the underlying mechanisms are, however, not understood in full detail. In particular, studies directly comparing the two non-verbal types of enrichment provided unclear results. Moreover, previous works often focused on examining effects of enrichments for one word type, predominantly nouns, sometimes differentiating between abstract and concrete nouns. An exception is a recent study by Garcia-Gamez and Macizo (2019) in which a comparison of the effects of different types of gestures on acquisition of different types of words has been conducted. Therefore, in the present study, we go beyond the majority of previous work by considering the role of different word types for their effectiveness while comparing the effects of pictures vs. gestures as non-verbal aids in L2 word learning. Further, we employ a virtual tutor in our study to enable for perfect control of bodily behavior. Also, this allows to transfer our finding easily onto technology-enriched learning environments, thus, informing the design of tutoring systems.

### 1.1. Effects of Picture-Based Enrichment

The use of pictures to support vocabulary acquisition is prevalent in L2 classrooms as well as online learning environments, such as DuoLingo or Babbel. Also, multiple scientific studies have been conducted, investigating the question whether pairing an image with text can improve word learning more than text alone.

We start with a review of studies in which participants' learned words in their native language that were unfamiliar or novel for them. Bull and Wittrock (1973) investigated the effect of image-based enrichment on learning novel English nouns. Ten-years-old students were taught in one of three conditions: verbal definition only, given imagery (an illustration had to be traced by participants), or self-discovered imagery where students had to draw their own illustrations of the definitions. Results indicated that students learned words better under the imagery conditions. In a study with 8-years-old children, McGregor et al. (2007) found that children performed better in a post-test definition task and picture-naming task after learning unfamiliar English words with richer semantic information, i.e., children learning the novel words with a definition plus picture were better in recall than those learning with definitions only.

Similar results can be found in studies with adult learners. In a study by Smith et al. (1987) undergraduate students performed better in a delayed post-test on learned novel English vocabulary when they received picture-enriched definitions for novel words comparison with definition-only, or definition plus the word used in a context sentence. Later, Smith et al. (1994) demonstrated in two more studies that students receiving novel English word with illustrations scored significantly higher on both immediate and delayed recall tests than students who were not provided with illustrations. Moreover, the study revealed individual differences to be relevant for the effectiveness of the interventions under investigation.

One of the few existing studies addressing the role of visual enrichment in the acquisition of L2 words has been conducted by Plass et al. (1998). English-speaking college students learning German as L2 read a German language story and could choose to view a picture or video clip representing the word or to see a translation on the screen in English, or a combination of both for particular keywords in the story. Word translations were better memorized when students had selected both visual and verbal annotations during learning than only one or no annotation.

A theoretical framework to explain the above-reviewed evidence of picture-based support for word learning, is the *Dual Coding Theory* (DCT, Paivio, 1971, 1986, 2007; Sadoski, 2005). DCT states that cognition involves the cooperative activities of two functionally independent, but interconnected systems: a verbal system specialized for dealing with linguistic information (in several modalities language, e.g., hearing or reading) and a non-verbal system specialized for dealing with non-linguistic information (e.g., objects and events). Connections within and between the systems are assumed to account for meaning and memory. Empirical support for DCT comes from neurophysiological studies on brain functions during language processing (cf. Shen, 2010). Di Virgilio and Clarke (1997) showed that there are parallel pathways in visual-verbal processing in one hemisphere. That is, concrete words that produce imagined visual images are processed simultaneously in verbal and non-verbal codes. Fiebach and Friederici (2004) showed that the processing of concrete vs. abstract words activates different areas in the brain's left hemisphere such that concrete words, that are associated with an image whereas abstract words are not, activate high-level visual areas of the left basal temporal lobe involved in mental image formation more than abstract words do. By contrast, abstract words activate the inferior frontal areas involved in the strategic retrieval of semantic information more strongly. Further behavioral evidence for DCT comes from observations that concrete words are processed faster and more accurately than abstract words in a variety of cognitive tasks (Jessen et al., 2000; Malhotra et al., 2001; Levy-Drori and Henik, 2006). The crucial claim of DCT in order to explain the supportive role of pictures in L2 vocabulary learning is based on the role of the connection of the two systems in information processing (Sadoski, 2005): According to DCT, the more one can activate both the verbal and non-verbal code while learning a new word, the better the word will be learned. As pictures can activate the non-verbal code, while words activate the verbal code, improved learning outcome is predicted for input consisting of word(s) and picture.

### 1.2. Effects of Gesture-Based Enrichment

There is a growing body of evidence suggesting that iconic gestures also bear a great potential to enhance learners' memory performance for novel words (Macedonia and Klimesch, 2014). The first systematic study on this topic was conducted by Quinn-Allen (1995): English-speaking students learned French expressions that were simultaneously accompanied by emblematic gestures. Participants who saw and imitated these gestures achieved higher memory recall rates than participants in a control group. Later studies replicated and extended these initial findings (e.g., Kelly et al., 2009; Macedonia and Knösche, 2011; Macedonia et al., 2011; de Nooijer et al., 2013, 2014; de Wit et al., 2017; Andrä et al., 2020; Sweller et al., 2020).

To exclude, first of all, an attentional effect of gestures, some studies compared meaningless or incongruent gestures against gestures semantically congruent with the words to be learned. The results of these studies demonstrate that (i) only congruent gestures benefit recall while incongruent gestures negatively affect recall (Kelly et al., 2009), and (ii) meaningful iconic gestures facilitate learning of L2 concrete nouns resulting in better recall compared to meaningless gestures (Macedonia and Knösche, 2011). In the latter study by Macedonia and Knösche (2011), functional magnetic resonance imaging revealed that words supported by iconic gestures during learning elicited greater signal intensity in the dorsal medial premotor cortex bilaterally during recognition and retrieval. This activation in the premotor cortices suggests that motor simulation processes are taking place during retrieval. Hence, the effectiveness of gesture-based enrichment might be due to the involvement of the motor system above visual and verbal memory. Similar evidence for overlapping regions in the motor cortex being activated at both learning and retrieval phases has been provided by other studies (Nilsson et al., 2000; Nyberg et al., 2001; Russ et al., 2003).

In sum, these neurocognitive findings support the *Motor Activation Hypothesis (MAH)* which states that the motor processes that take place during encoding are re-activated during retrieval with this critical activation of motor areas during recognition and recall from memory being the reason for improved memorization outcome. In fact, an investigation by Ianì and Bucciarelli (2017) showed that the beneficial effect of gesture observation on word learning only takes place when participants do not perform a secondary motor task that involves the same effectors parallel to observing the gestures. When the second task, however, involves other effectors than the gesture being observed, the gesture-benefit persists.

In studies with children, de Nooijer et al. (2013) investigated the effects of gesture observation vs. different variants of gesture imitation on children's learning of different types of novel L1 verbs (manipulation verbs, locomotion verbs, abstract verbs). The hypothesis that gesture imitation would lead to better recall was partially supported in that imitation benefitted the recall of the object-manipulation verbs only, but not for locomotion and abstract verbs. Given that the gestures used in the study were hand- and arm gestures, the results can still be interpreted in the sense of MAH, however, suggesting that the words to be learned need to have a link to the motor system that is activated by gestures. For abstract verbs one might argue that these lack a direct link to the motor system. For locomotion verbs, manual gestures might not be adequate to activate the relevant motor representations. In fact, a follow-up study by de Nooijer et al. (2014) in which full-body gestures were employed for locomotion verbs whereas manual action verbs were accompanied by hand-/arm gestures, revealed no major differences with respect to learning outcome when children observed gestures in the learning phase. The later study by de Nooijer et al. (2014) also evaluated gesture-based enrichment for different types of novel L1 verbs (object manipulation, locomotion and abstract verbs). In addition, the kind of instruction children received was also manipulated. Children were provided either with a verbal definition alone, or a verbal definition in combination with either gesture observation, imitation, or self-generated gestures. Results suggest that the effectiveness of gestures for vocabulary learning differs depending on verb type: Especially learning of locomotion verbs showed to be improved through gesture observation compared to verbal definitions only. In addition, there were differences according to children's language proficiency level. Children with good language skills could benefit from imitation and self-generated gestures for learning object-manipulation verbs, while this appeared to hinder children with poor language skills. Tellier (2005) describes an experiment in which children had to memorize words in L1 that were illustrated by gestures. Children who had imitated the gestures while learning memorized better than those who only observed gestures and than those of the control group.

Testing adult learners, Morett (2014, 2018) obtained similar results and showed that gesture production facilitates L2 words (Hungarian) recall more effectively than gesture viewing. In sum, gesture-based benefits on memorizing linguistic materials occur both when participants perform the gestures themselves and also when they just observe the gestures produced by others (cf. Ianì and Bucciarelli, 2017; Sweller et al., 2020). Some studies even found memorization to be better when the motor system is engaged via self-performed action as opposed to observed action, i.e., when learners are engaged in gesture imitations vs. gesture observation.

One major finding of the reported studies was that gesture-based enrichment in word learning depends on the type of linguistic material to be learned (de Nooijer et al., 2013, 2014). Evidence along the same lines has been provided by Macedonia et al. (2011) who conducted a study in which they focused on a comparison of word types (concrete and abstract nouns, verbs, and adverbs). Overall, gesture-enrichment (imitation of gesture) outperformed a speech-only control condition for all word classes under investigation. Regarding the effect of word type, the results showed that concrete nouns were best memorized, followed by verbs, abstract nouns, and adverbs. Also, an interaction effect between word type and training emerged such that concrete nouns could profit most from gesture-based enrichment as opposed to abstract nouns. This finding can also be interpreted in the sense of the MAH as well as DCT as it seems intuitive that concrete words are easier to relate to sensory, motor or imagistic information than other words (cf. Sadoski, 2005). Another study supporting the claim that gesture-based enrichment in word learning depends on the type of linguistic material to be learned has been conducted by Garcia-Gamez and Macizo (2019) who evaluated the impact of gestures on L2 vocabulary learning with both nouns and verbs. Better vocabulary learning was found for both word types when participants learned L2 words with congruent gestures relative to a no gesture condition, and further, the use of gestures in L2 vocabulary acquisition appeared to remediate the intrinsic difficulty associated with the learning of verbs.

Finally, first steps have been taken to transfer the findings on gesture-supported vocabulary learning toward artificial agents (virtual humans or humanoid robots). Bergmann and Macedonia (2013) were among the first to compare a control group with no gestures to the effects of iconic gestures performed by a real human or a virtual human as input for learners, respectively. Overall the results showed improved recall (immediate and delayed) when participants learned with the virtual human than when learning in the control condition. By trend, the virtual human condition also outperformed the human trainer. Especially high performers could profit from gesture-supported training with the artificial agent. Others research groups used a social robot to teach learners novel vocabulary. van Dijk et al. (2013) used a Nao robot (a small humanoid robot designed to interact with people, cf. https://softbankrobotics.com/) that provided older adult learners (55+) with short verbal messages that were supported by either iconic gestures or no gestures while establishing eye contact with the participant or averting eye contact. The gestures depicted the action described in the verb of the sentence. While eye contact did not affect learning, the use of iconic gestures benefited retention of the verb to which the action-depicting gestures pertained. de Wit et al. (2017, 2018, 2020) also employed a Nao robot to teach Dutch children English animal names. They found in a post-test on children's comprehension of the L2 words a significant benefit of gesture use over the no-gesture condition in two of their studies (de Wit et al., 2017, 2018), but no positive effect of iconic gestures in a third study (de Wit et al., 2020). Lan et al. (2018) examined the effect of production of motions associated with sports via learners' own bodies or avatars on ESL sports vocabulary learning. The results depict that students learned better by watching their own 3D avatars doing motions than by moving their own bodies to produce the motions or doing nothing.

### 1.3. Picture-Based vs. Gesture-Based Enrichment

A couple of studies have been conducted to directly compare picture-based and gesture-based enrichment in novel word memorization. Most of these studies have been conducted with children as participants.

Several studies demonstrate the benefit of learning with gestures as compared to pictures. For instance, McGregor et al. (2009) examined 2-years old children with respect to comprehension of the spatial term under. Children either viewed a symbolic gesture for under during training, or they viewed a still photograph of objects in the under relationship, or they did not receive any supplemental symbolic support. Children's knowledge of under was measured at three time points: directly before, immediately after, and 2–3 days after the training. A gesture advantage emerged for the delayed post-test. While McGregor et al. (2009) focused on comprehension, Kapalková et al. (2016) tested 2-years-old on word production. Children were taught novel words that were either accompanied by a picture or by a gesture. Production tests took place immediately after training, at a 2-weeks follow-up and at a 6-weeks follow-up. The results show that gesture training was beneficial over picture training and supported word learning significantly across all three testing points. Moreover, Tellier (2005) worked with mono-lingual French children (5–6 years) who had to learn L2 words (English), whereas while half of the children were taught with pictures, while the other half of the children were taught with accompanying gestures that had to be imitated. Results showed that the gesture group performed significantly better than the picture group in the recall.

Other studies reproduced mixed results. In this regard, Khanukaeva (2014) found that second grade pupils who were presented with English L2 words paired with either an iconic gesture or an image did not benefit from using gestures over pictures in the immediate recall test. The delayed post-test, however, revealed that learners could benefit significantly from the gesture-based enrichment vs. picture-based enrichment in the long term. A study by Rowe et al. (2013) did not find support for a benefit of enrichment conditions (picture, gesture) over the control condition with enrichment on the recall of pseudo-words for familiar objects. However, pictures' and gestures' helpfulness varied as a function of the learners' gender and language ability. Girls performed better than boys on the translation task when they had learned with pictures. And children with low verbal abilities had a higher benefit from learning with gestures. Overall, these results suggest that considering the interplay between learner characteristics and instructional strategies is important. In a recent study, Andrä et al. (2020) demonstrated the benefit of gestures over a control condition (auditorily presented words only) on children's retention of abstract and concrete nouns in two studies. A third study solely comparing pictures and gestures (without control), however did not reveal a benefit of gestures over pictures (Andrä et al., 2020).

The studies reviewed so far, compared gesture- vs. picture-based enrichment of learning (mostly) novel L1 words, in children at different age groups, mostly. There are, however, fundamental differences between children learning new L1 words and adults learning L2 words. Adults already have a well-established conceptual and lexical L1 system that is actively involved in the L2 learning process, whereas children learn words and concepts at the same time (Jiang, 2004). Studies with adult learners comparing gestures and pictures are rare. Repetto et al. (2017) conducted a study with twenty young adults who learned novel abstract words either by reading only or reading and pairing the novel word to a picture or, lastly, reading and enacting the word by means of a gesture. In a recognition task participants made less errors for words encoded with gestures compared to words encoded with pictures. Similarly, Mayer et al. (2015, 2017), compared gestures and pictures with regard to their enrichment effect in L2 vocabulary learning with adults. Participants learned L1–L2 word pairs (concrete and abstract nouns) whereby an artificial foreign language with Italian phonotactics was employed. Mayer et al. aimed to address the role of different motor tasks for the enrichment effect and set up two versions of their experiment. In experiment 1, participants imitated gestures congruent with the word meaning, and in the picture-enriched condition, they copied the outline of a picture illustrating the word meaning. In a control condition, participants learned without any enrichment. In experiment 2, the same materials were employed, but participants did not perform any motor tasks. That is, in the two enrichment conditions of experiment 2, the stimuli were simply observed. In addition, the two versions of the experiment yielded different results regarding the effectiveness of non-verbal enrichment. In experiment 1 (with motor tasks) gestures outperformed pictures. In experiment 2 (without motor tasks), however, pictures outperformed gestures (Mayer et al., 2015). Similarly, Morett (2019) demonstrated that L2 learners of Hungarian benefited most from viewing picture-enhancement when learning concrete words in comparison to glosses and viewing iconic gestures, but Morett only tested viewing conditions and no enactment conditions. Are gestures particularly strong when they are performed whereas pictures deploy their effectiveness when being viewed? A transcranial magnetic stimulation study by Mathias et al. (2020) might provide support for this hypothesis. The results of this study showed that when repetitive transcranial magnetic stimulation (rTMS) was applied to the bilateral posterior motor cortices participants' translation slowed down for those L2 words that had been learned with gesture enrichment, but not for words learned with picture enrichment.

### 1.4. The Current Study

In summary, the above review of literature revealed that word learning can be improved by picture-based enrichment (Bull and Wittrock, 1973; Smith et al., 1987, 1994; Plass et al., 1998; McGregor et al., 2007) as well as gesture-based enrichment (Quinn-Allen, 1995; Kelly et al., 2009; Macedonia and Knösche, 2011; Macedonia et al., 2011; de Nooijer et al., 2013, 2014; Macedonia and Klimesch, 2014).

Comparison studies of gesture- vs. picture-based enrichment predominantly addressed children as learners. The majority of these studies found gestures to be more effective than pictures—at least when measured with delayed post-tests (Tellier, 2005; McGregor et al., 2009; Khanukaeva, 2014; Kapalková et al., 2016). Rowe et al. (2013), however, found no clear benefit of pictures and gestures over a control baseline. Studies with adult learners are sparse and results do not clearly speak for or against one of the enrichment techniques, suggesting that the way how learners deal with the picture-/gesture input might be essential (Mayer et al., 2015, 2017; Repetto et al., 2017; Morett, 2019; Andrä et al., 2020). Moreover, some works indicate that interindividual differences (Rowe et al., 2013; de Nooijer et al., 2014) as well as word types to be learning (Macedonia et al., 2011; de Nooijer et al., 2013) play a significant role in L2 vocabulary learning.

The current study, therefore, extends this line of research and provides a direct comparison of imitating a semantically congruent gesture vs. observing a semantically congruent picture on adults' acquisition of L2 words. Given the practical relevance of this question for designing digital learning environments equipped with pedagogical agents, we employ a virtual character instead of a human to perform the gestures. According to the MAH, we hypothesize that gesture imitation leads to stronger activation of motor representations associated with the linguistic material than picture observation.

H1: Both types of non-verbal enrichment (pictures and gestures) are more effective than no enrichment with regard to word learning measured in immediate (H1a) and delayed (H1b) recall tests.
H2: Gesture-based non-verbal enrichment is more effective than picture-based non-verbal enrichment with regard to word learning measured in immediate (H2a) and delayed (H2b) recall tests.

Given that previous research found differences in the effectiveness of non-verbal aids for different word types (Macedonia and Knösche, 2011; de Nooijer et al., 2013, 2014) we investigated different word categories and sk:

RQ1: What is the influence of word classes (noun, verb, adjective) on the effectiveness of non-verbal enrichment types in word learning?
Our hypotheses state that enrichment benefits word learning (H1) with gesture-based enrichment benefiting the most (H2). However, this assumed effect might not only be affected by word class but also might show differences with regard to the language to be learned (L1 words or L2 words) as well as with regard to the test interval (immediate vs. delayed):
RQ2: Is the effectiveness of non-verbal enrichment types on word learning different taking into account the language of the words to be learned (L1 words, L2 words, L1–L2 pairs)?
RQ3: Is the effectiveness of non-verbal enrichment types on word learning different taking into account the test interval (immediate vs. delayed recall)?
Given that previous research found differences in the effectiveness of non-verbal aids based on learner's individual differences (Rowe et al., 2013; de Nooijer et al., 2014) we tested participants for their preferences in learning style and pose the following question:
RQ4: What is the influence of individual language learning styles (visual learner, auditory learner, kinesthetic learner) on the effectiveness of non-verbal enrichment types in word learning?

## 2. Method

In 3 × 3 within-subjects design we manipulated the kind of enrichment: gesture imitation, picture observation, and no enrichment as control. In addition, three different word classes were addressed: nouns, verbs, and adjectives. Participants trained German-Finnish vocabularies on 3 consecutive days. Their immediate learning performance was measured the next day prior to the next training session (day 2, day 3) and on day 4 without a training following. The long-term effect of information decay was measured additionally with a delayed measure 4 weeks after training (day 28).

### 2.1. Materials

The training materials comprised 45 German-Finnish word pairs (15 nouns, adjectives, and verbs, respectively). So, in contrast to Mayer et al. (2015, 2017), our study employed natural L2 materials instead of artificial linguistic items. Despite the advantages of an artificial language in terms of avoiding any kind of pre-knowledge learners might have, using a typologically different language with learners who do not have any previous experience with that language type (as Finnish is for German natives) fulfills the same purpose while at the same time exhibiting the characteristics of a naturally evolved language.

The items to be learned have been controlled for word length across experimental conditions for the Finnish words. Results showed no differences in the amount of syllables per word across word types [*F*_(2)_ = 0.272, *p* = 0.374] and across enrichment conditions [*F*_(2)_ = 1.006, *p* = 0.763]. In addition, the experimental conditions were matched for lexical frequency of the German words using a word frequency counter of German. Frequency is comparable across word classes [noun, verb, adj; *F*_(2)_ = 0.393, *p* = 0.677] and across enrichment conditions [gesture, picture, control; *F*_(2)_ = 0.788, *p* = 0.462].

Moreover, the level of concreteness, imageability and familiarity of the words was assessed for each category with the MRC psycholinguistic database (Coltheart, 1981). As the MRC database is available for English only, the German/Finnish terms were translated into English words to that end. Results showed that the words used in our three respect training conditions were comparable in their level of concreteness [*F*_(2)_ = 0.016, *p* = 0.984], imageability [*F*_(2)_ = 1.454, *p* = 0.245], and familiarity [*F*_(2)_ = 0.158, *p* = 0.854]. Likewise, the items of the three word classes were comparable in their level of concreteness [*F*_(2)_ = 0.889, *p* = 0.419], imageability [*F*_(2)_ = 0.407, *p* = 0.668], and familiarity [*F*_(2)_ = 2.190, *p* = 0.125].

**Figure 1:**
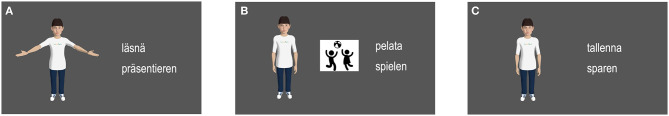
Examples for stimulus presentation in the three experimental conditions: **(A)** Gesture-based enrichment for “to present” (Fin.-Ger.: läsnä—präsentieren); **(B)** picture-based enrichment for “to play” (Fin.-Ger.: peleta—spielen); **(C)** no enrichment for to scrape (Fin.-Ger.: tellenna—sparen).

A virtual human was present on the screen together with written word pairs in all three conditions to avoid any kind of presence or persona effect which might take place with the virtual human only being present for gesture-enriched items (cf. Lester et al., 1997). For the picture-enriched items, written word pairs were accompanied by a picture matching the word pair semantically. Gesture-enriched items were accompanied by a semantically congruent gesture performed by a virtual human to be imitated by participants. The virtual human's gestures were specified in the Behavior Markup Language (BML; Vilhjálmsson et al., 2007) and realized with the ASAP behavior realizer (Kopp et al., 2014), an architectural framework for conversational agents enabling fluid real-time conversation. All stimuli were rendered into video data, for example screenshots, see Figure 1.

For both, gestures and pictures, a pre-test has been carried out to evaluate whether the enrichments matched with the linguistic items semantically. To this end, an online study was conducted in which German participants (*N* = 14) were provided with the gesture video or the picture and the German word. Their task was to judge in how far word and respective gesture/picture were matching on a 7-point Likert scale (1: not at all; 7: perfectly). Ratings of semantic congruence for the two enrichment conditions were comparable [*t*_(13)_ = 0.792, *p* = 0.442]. For gestures performed by the virtual human the mean of participants' ratings was 4.47 (*SD* = 0.39, min = 3.67, max = 5.67), for pictures the mean of ratings was 4.63 (*SD* = 0.82, min = 3.0, max = 6.27). The final items used in this study were the following. All non-verbal enrichments-pictures and videos of gestures- can be made available. The gestures can be found in the Supplementary Material (Data Sheet 2). The pictures are available upon request via the contact author.

Word pairs used for *control condition* without any enrichment:

- nouns: Erfahrung-kokemus (experience); Freiheit-vapaus (freedom); Freundschaft-ystävyys (friendship); Sport-urheilu (sports); Wahrheit-totuus (truth);
- verbs: etablieren-perustaa (establish); gestalten-muoti (create); kritisieren-arvostella (criticize); organisieren-järjestää (organize); sparen-tallenna (save)
- adjectives: aufmerksam-huomiota (attentive); bekannt-tiedossa (known); direkt-suora (direct); gesund-terve (healthy); treu-uskollinen

Word pairs used for the *gesture-enrichment condition*:

- nouns: Angst-pelko (fear); Geheimnis-salaisuus (secret); Glaube-usko (faith); Kraft-voimassa (strength); Liebe-rakkaus (love)
- verbs: denken-ajatella (think); entspannen-rentoutua (relax); präsentieren-läsnä (present); träumen-unelma (dream); verbinden-liitä (unite)
- adjectives: fern-kauko (distant), laut-mukaan (loud); müde-väsynyt (tired); spät-myöhään (late); verboten-kielletty (forbidden)

Word pairs used for the *picture-enrichment condition*:

- nouns: Einheit-yksikkö (unity); Forschung-tutkimus (science); Kommunikation-viestintä (communication); Orientierung-ssunta (orientation); Zentrum-keskus (center)
- verbs: helfen-apua (help); lernen-oppia (learn); spielen-pelata (play); suchen-etsi (search); vergessen-unohda (forget)
- adjectives: bequem-kätevä (comfortable); erfolgreich-onnistunut (successful); glücklich-iloinen (happy); krank-sairas (sick); langsam-hidas (slow)

### 2.2. Participants and Procedure

A total of 32 native speakers of German participated in the study. Six participants did not complete all five study appointments. Their incomplete data were omitted for the analyses. The remaining 26 participants included in the study were aged from 18 to 36 (*M* = 24.2, *SD* = 4.4), 19 participants were female and seven participants were male. None of the participants had prior knowledge of the Finnish language. All of them were paid €40 for their participation. We assessed participants' individual language learning style using the Learning Style Survey by Cohen et al. (2001), based on Oxford (1990, 1995) and Ehrman et al. (2003). Participants filled in items asking for preferences regarding visual, auditory or tactile/kinesthetic learning styles. Unfortunately, with Cronbach's alpha values between 0.403 (for visual sensory preferences) and 0.621 (for hands-on preferences) the scores did not reach a satisfying level of internal validity. Therefore, we did not include the individual language learning style into further analyses.

Upon arrival on the first day participants read and signed informed consent. Participants were informed that they took part in a study on foreign language vocabulary learning with the goal to memorize as many words as possible. Participants were also informed that their performance would be assessed at different time points through different kinds of written tests. The training lasted ~45 min per day on 3 consecutive days. After the three training days participants were additionally invited on the 4th and 28th day for recall tests.

Training was performed in groups of five to ten participants, respectively. The total of 45 words were subdivided into three blocks of 15 words each, in which the three training conditions alternated daily and counterbalanced the experimental conditions. In each block, the items were subdivided into three smaller blocks of five items out of one word class each. [cf. Supplementary Material (Data Sheet 4) for a plan of procedures].

For each item the learning materials appeared step by step such that the Finnish word was shown alone for 5,000 ms (audio file of pronunciation was played after 2,000 ms). Subsequently the German translation was added (again, an audio file of the pronunciation was played after 2,000 ms). Another 5,000 ms later, the enrichment was presented for 5,000 ms, resulting in a total duration of 15 s per sequence which was presented three times in succession. Prior to the first sequence, learners were instructed to watch the materials. Prior to second and third sequence, learners were instructed to speak the Finnish word synchronously (a short audio signal was played to inform participants about the start of the vocalization) and to imitate the gestures (only for items with gesture-based enrichment). After each training block consisting of 15 items, a 5 min break followed before the next block started [cf. Supplementary Material (Data Sheet 4) for a plan of procedures].

Immediate memory performance was assessed daily starting from the second experiment day measuring the learning outcome of the first training day etc. (three points of measurement: day 2, day 3, day 4). On day 4, an assessment of participants' language learning style took place using the Learning Style Survey (Cohen et al., 2001). Finally, a delayed test of memory performance has been conducted on day 28. Participants were administered a free recall test in which they were provided with an empty sheet. They were instructed to write as many items as possible in both languages. Written test was used in order to be able to spell check participants answers. Items could be loose (i.e., only German or only Finnish) or matched (i.e., Finnish and German). Items recalled in a pair also counted into the loose recall. Items were considered correct if their spelling corresponded 100% to the word spelling provided during training or in case of minor mistakes, e.g., interchanged letters like pelko-peklo or single instead of double letters like tallena-tallenna for the Finnish words or in case of nominalization effects like to play-play in German. Recall rates (in percentage) were calculated for all word types separatedly.

After conclusion of the study, participants were thanked for participation, received their compensation and were debriefed about the study.

## 3. Results

The collected data has been analyzed for participants' recall rates of the taught German-Finnish words pairs in L1, in L2, and in L1–L2 word pairs, separately. To test if enrichment type, word type, and test interval were related to recall rates of L1 and L2 words as well as L1–L2 pairs. We used R (R Core Team, 2020) and lme4 (Bates et al., 2015), which allowed for mixed-effects modeling. Three linear mixed-effects models predicted L1 recall, L2 recall, and L1–L2 pairs recall separately.

### 3.1. Recall of L1 Words

**Figure 2:**
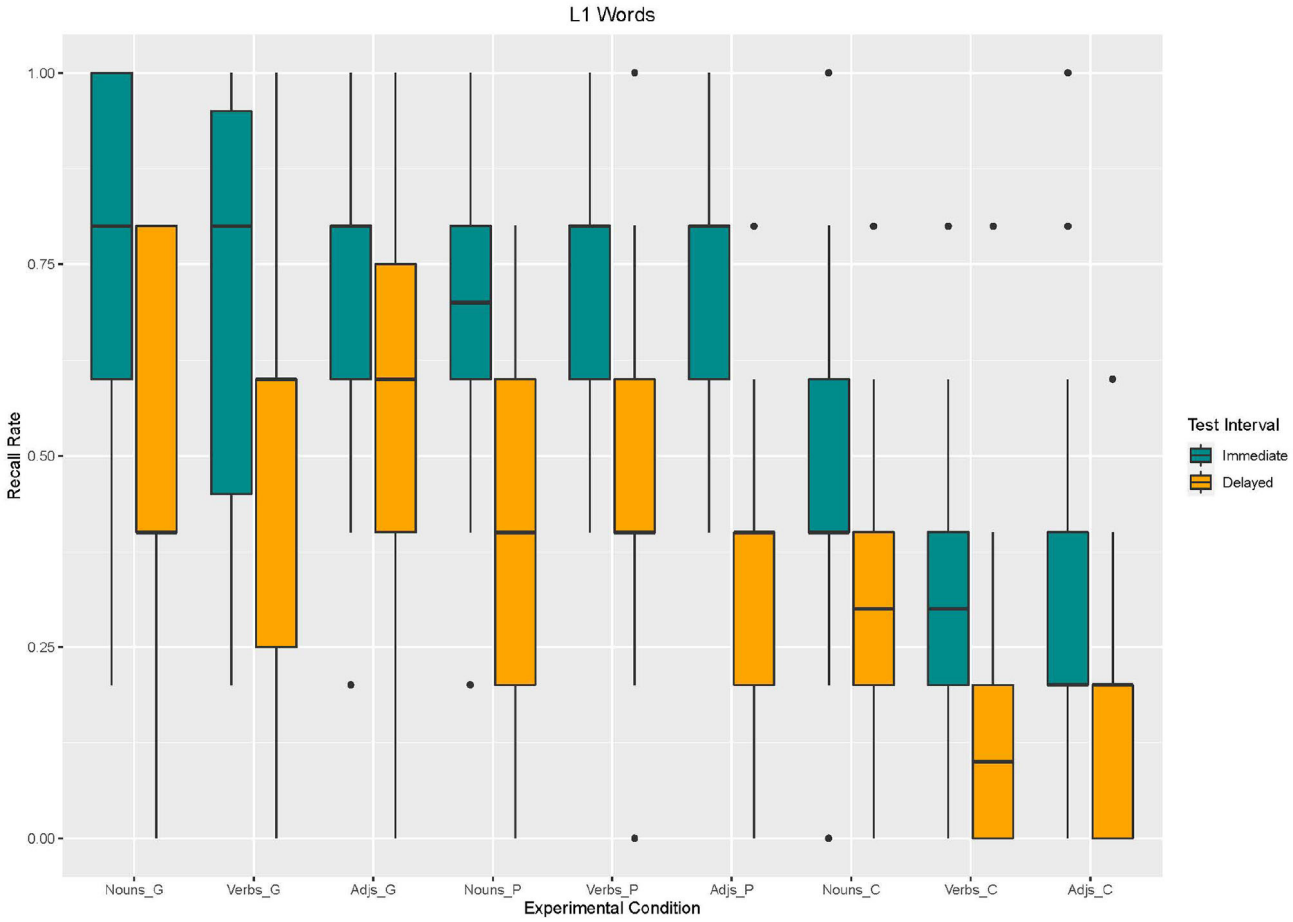
Box Plots of Recall rates of L1 words as a function of enrichment type and word type in immediate and delayed test interval.

**Figure 3:**
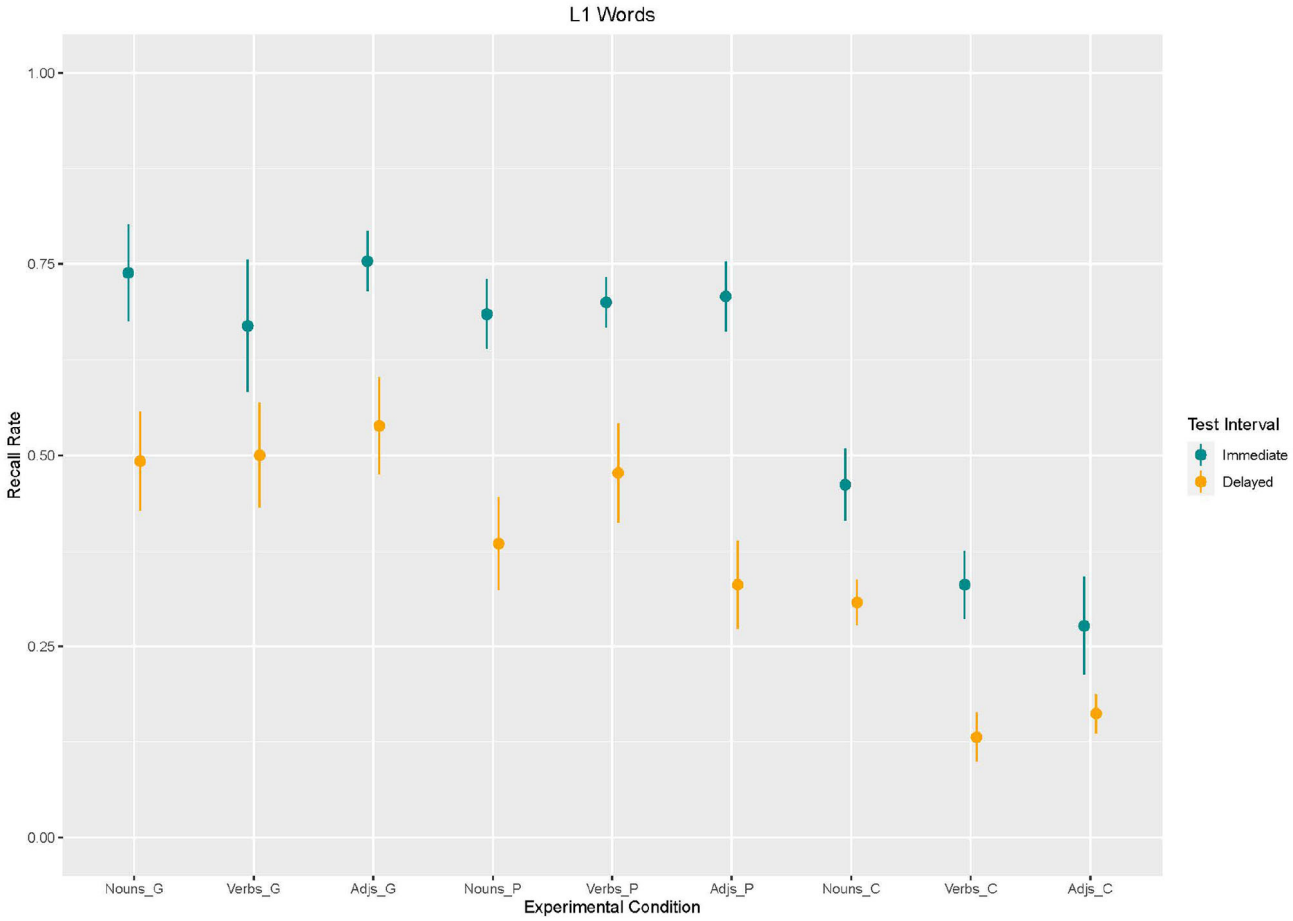
Mean values and variance of Recall rates of L1 words as a function of enrichment type and word type in immediate and delayed test interval.

We performed a linear mixed effects analysis of the relationship between recall of L1-words and enrichment type, word type, and test interval. As fixed effects, we entered enrichment type (coded: 1 = gesture, 2 = picture, 3 = control), word type (coded: 1 = noun, 2 = verb, 3 = adjective), and test interval (1 = immediate, 2 = delayed). In three different models we compared the influence of different random effects. Model 1 included intercepts for participants; model 2 included intercepts for participants and items; and model 3 included intercepts for participants and by-item random slopes for participants. Model comparison showed that model 3 fitted best (cf. Table 1). Based on Model 3, we found an effect of *enrichment type*, with lower recall more likely to occur in the control condition than in the gesture condition [β = −276.9, *SE* = 55.63, *z*_(2340)_ = −4,978, *p* < 0.001]. Moreover, an effect for *test interval* was found with lower recall more likely to occur in the delayed recall than in the immediate recall [β = −246.2, *SE* = 55.63, *z*_(2340)_ = −4,424, *p* < 0.001]. No main effect for *word type* emerged. However, analysis showed an *interaction effect* between word type and enrichment type as to that adjectives without any enrichment (control condition) were recalled less often than nouns supported by gestures [β = −200, *SE* = 78.68, *z*_(2340)_ = −2,542, *p* = 0.01; cf. Figures 2, 3].

**Table 1:** Comparison of three linear mixed effects models: Model 1 with intercepts for participants, Model 2 with intercepts for participants and items, Model 3 with intercepts for participants and by-item random slopes for participants.

		**Df**	**AIC**	**BIC**	**logLik**	**Deviance**	**χ^2^**	**χ^2^Df**	***p***
L1 words	Model 1	20	2960.5	3075.6	−1460.2	2920.5			
	Model 2	21	2953.8	3074.7	−1455.9	2911.8	86.69	1	0.003
	Model 3	21	2953.6	3074.5	−1455.8	2911.6	0.24	0	<0.001
L2 words	Model 1	20	2072.2	2187.3	−1016.1	2032.2			
	Model 2	21	2053.5	2174.4	−1005.8	2011.5	206.24	1	<0.001
	Model 3	21	2050.7	2171.6	−1005.9	2008.7	28.44	0	<0.001
L1–L2 words	Model 1	20	1941.5	2056.7	−950.76	1901.5			
	Model 2	21	1919.4	2040.3	−938.69	1877.4	24.14	1	<0.001
	Model 3	21	1908.1	2029.0	−933.05	1866.1	11.28	0	<0.001

### 3.2. Recall of L2 Words

We used the same model specifications for the analysis of L2 words as for the L1 words and performed a linear mixed effects analysis of the relationship between recall of L2-words and enrichment type, word type, and test interval (fixed effects) and compared the influence of different random effects. Again, the model comparison showed that model 3 fitted best (cf. Table 1).

**Figure 4:**
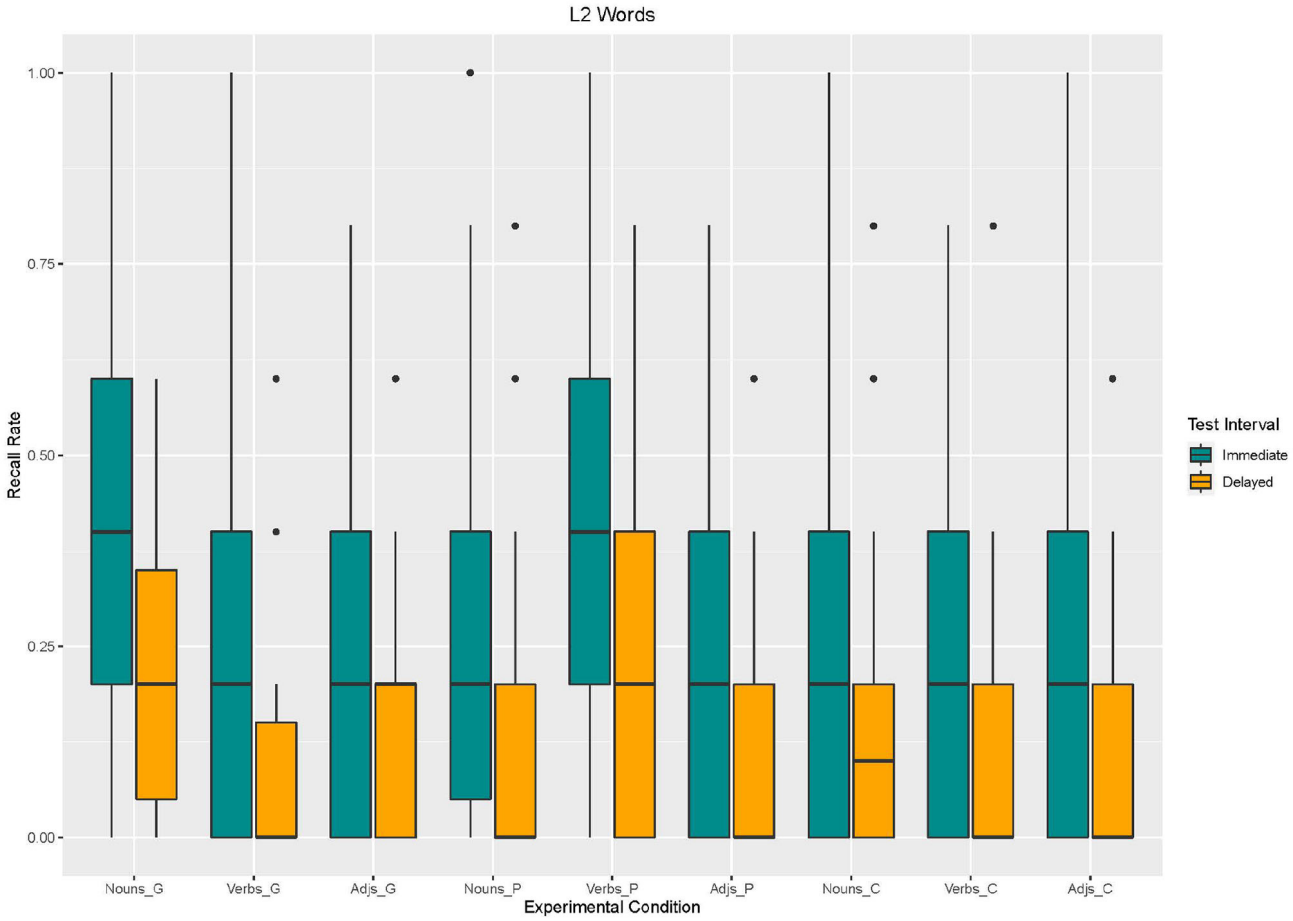
Box Plots of Recall rates of L2 words as a function of enrichment type and word type in immediate and delayed test interval.

**Figure 5:**
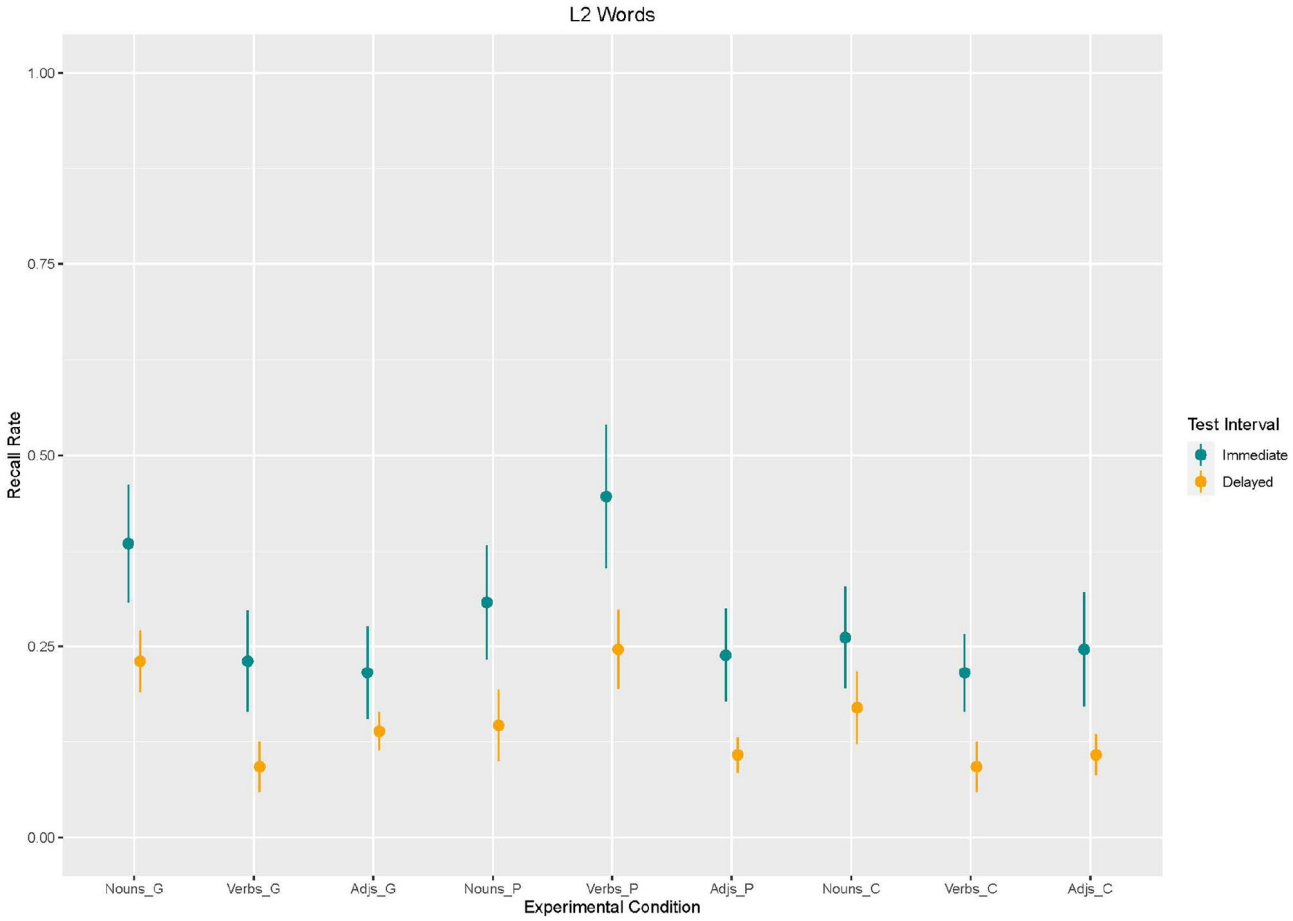
Mean values and variance of Recall rates of L2 words as a function of enrichment type and word type in immediate and delayed test interval.

Based on Model 3, we found an effect of *enrichment type*. Recall in the control condition [β = −123,1, *SE* = 44.91 *z*_(2340)_ = −2,740, *p* < 0.001] and in the picture condition [β = −76.92, *SE* = 44.91 *z*_(2340)_ = −1,713, *p* < 0.09] was lower than in the gesture condition. *Word type* also had an effect on recall. Recall for verbs [β = −153.8, *SE* = 44.91 *z*_(2340)_ = −3,426, *p* < 0.001] and adjectives [β = −169.2, *SE* = 44.91 *z*_(2340)_ = −3,768, *p* < 0.001] was lower than for nouns. Moreover, an effect for *test interval* emerged. Recall was lower in the delayed recall than in the immediate recall [β = −153.8, *SE* = 44.91, *z*_(2340)_ = −3,426, *p* < 0.001]. With regard to *interaction effects*, the analysis revealed interaction effects between word type and enrichment type (cf. Figures 4, 5). Similarly to L1 words results, adjectives and verbs without any enrichment (control condition) were recalled less often than nouns supported by gestures [adjectives: β = 153.8, *SE* = 63.52, *z*_(2340)_ = 2,422, *p* = 0.02; verbs: β = 107.7, *SE* = 63.52, *z*_(2340)_ = 1,696, *p* = 0.09]. Moreover, verbs supported by pictures were better recalled than nouns supported by gestures [β = 292.3, *SE* = 63.52, *z*_(2340)_ = 4,602, *p* < 0.001].

### 3.3. Recall of L1–L2 Word Pairs

We used the same model specifications for the analysis of L2 words as for the L1 words and performed a linear mixed effects analysis of the relationship between recall of L2-words and enrichment type, word type, and test interval (fixed effects) and compared the influence of different random effects. Also for the L1–L2 word pairs the model comparison showed that model 3 fitted best (cf. Table 1).

**Figure 6:**
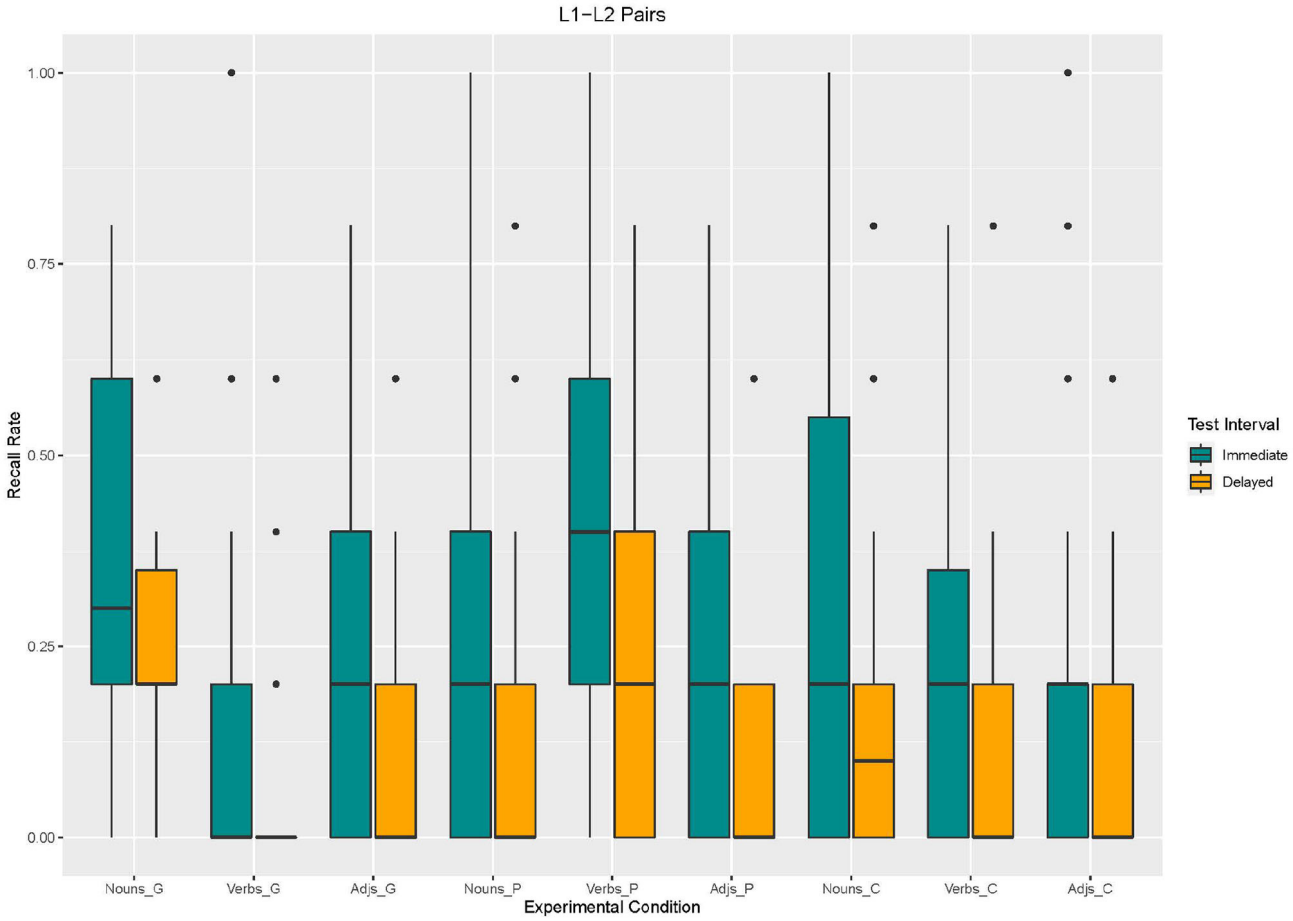
Box Plots of Recall rates of L1–L2 word pairs as a function of enrichment type and word type in immediate and delayed test interval.

**Figure 7:**
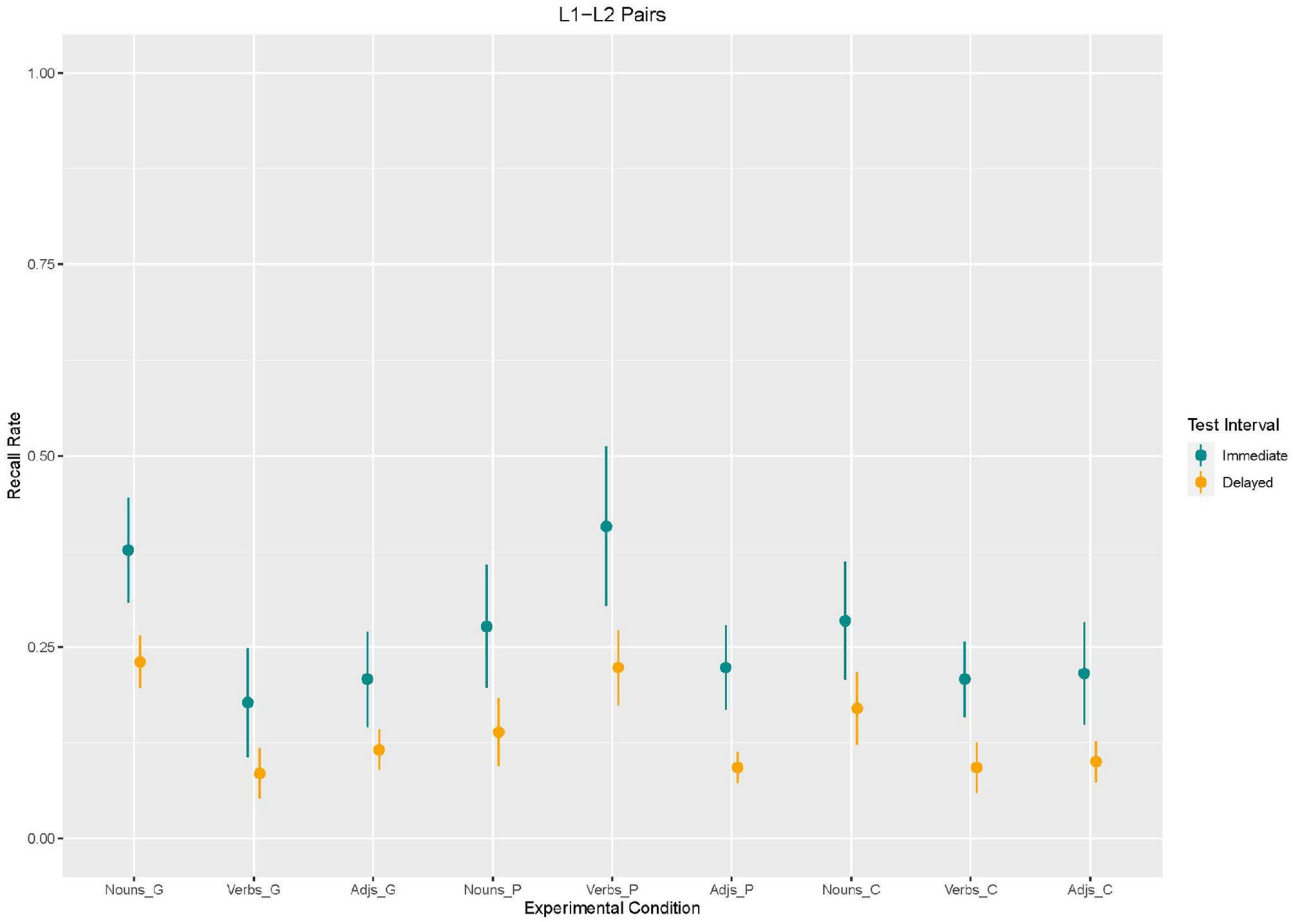
Mean values and variance of Recall rates of L1–L2 word pairs as a function of enrichment type and word type in immediate and delayed test interval.

Using Model 3, we found an effect of *enrichment type*. Recall in the control condition [β = −92.31, *SE* = 43.43 *z*_(2340)_ = −2,126, *p* = 0.03] and in the picture condition [β = −100, *SE* = 43.43 *z*_(2340)_ = −2,303, *p* = 0.02] was lower than in the gesture condition. The effects for *word type* suggest that verbs and adjectives were less recalled than nouns [verbs: β = −200, *SE* = 43.43 *z*_(2340)_ = −4,605, *p* < 0.001; adjectives:β = −169.2, *SE* = 43.43 *z*_(2340)_ = −3,897, *p* < 0.001]. Moreover, an effect for *test interval* emerged. Recall was lower in the delayed recall than in the immediate recall [β = −146.2, *SE* = 43.43, *z*_(2340)_ = −3,366, *p* < 0.001]. With regard to *interaction effects*, the analysis revealed interaction effects between word type and enrichment type (cf. Figures 6, 7). Adjectives and verbs supported by picture enrichment differed from gesture-based enrichment for nouns with verbs being better recalled and adjectives being less recalled [adjectives: β = 330.8, *SE* = 61.41, *z*_(2340)_ = 5,386, *p* < 0.001; verbs: β = 115.4, *SE* = 61.41, *z*_(2340)_ = 1,879, *p* = 0.06]. Moreover, verbs without any enrichment (control condition) were less recalled than nouns supported by gestures [β = 123.1, *SE* = 61.41, *z*_(2340)_ = 2,004, *p* = 0.04].

## 4. Discussion

The aim of the present study was to explore the role of non-verbal enrichment in L2 vocabulary learning by adding to the previous literature in several ways. First and foremost, it provides a direct comparison of enrichment through gesture imitation vs. picture observation with adult learners and for different word classes. Moreover, a virtual human was present in all conditions and provided learners with the gesture stimuli, making results directly relevant for the design of intelligent tutoring systems incorporating pedagogical agents. Finally, in contrast to Mayer et al. (2015, 2017), our study employed natural L2 materials instead of artificial linguistic items.

Our results indeed show to some extent the beneficial nature of non-verbal enrichment in vocabulary learning, however, our hypothesis (H1) was only partially supported since not all word types profited from non-verbal enrichments in immediate and delayed recall of L2 words (or L1–L2 pairs), i.e., adjectives were not affected. Our MAH-based prediction that gesture imitation should be more effective than picture observation due to stronger activation of motor representations associated with the linguistic material (H2) was partially supported. The effects turned out to differ as a function of word type (R1) and the kind of vocabulary knowledge being measured (RQ2). With regard to the influence of the test interval on the effectiveness of enrichment types in word learning (RQ3), we observed no such interaction but rather a general decrease in recall in the delayed recall. As concerns the results regarding RQ2, effects of non-verbal enrichment were different for L1 words vs. L2 words (as well as L1–L2 word pairs). Memorization of L1 words turned out to benefit from both gesture-based and picture-based enrichment regardless of word type, while gesture-based enrichment still outperformed pictures. Whereas, memory performance for L2 words showed mixed results: gesture-based enrichment facilitated the recall of nouns while picture-based enrichment had a very pronounced effect on verbs. Adjectives, however, did not benefit from non-verbal enrichment at all in L2 or L1–L2 word pairs recall. The fact that L1 words and L2 words are not learned equally well is most probably due to memorizing L1 words (memorizing a known concept including its known linguistic representation) being less challenging than memorizing novel L2 words (a known concept needs to be coupled to an unknown linguistic representation). In fact, there were similar effects in related studies (e.g., Macedonia and Knösche, 2011). More surprising, however, is the fact L1 words and L2 words were affected differently by different types of non-verbal aids and in relation to word types.

Our data showed that gestures were particularly beneficial for learning nouns, whereas for learning verbs, pictures were more helpful (RQ1). How can we explain this? One possibility could be that learners are differently challenged by memorizing words of different word classes. At least for children in L1 acquisition, a noun learning advantage (“noun bias”) has been observed for many languages (e.g., Gentner, 1982; Bornstein et al., 2004). This could also be shown in our study. For the L2 recall and L1–L2 pairs recall nouns were significantly better recalled than adjectives. Researchers proposed several explanations for this observation. The syntactic complexity argument, for instance, states that verbs are inherently more syntactically and conceptually complex than nouns (Akhtar et al., 2001; Tomasello, 2003). Alternatively, the noun-dependency hypothesis suggests that verbs are harder to learn because they require noun knowledge for their requisite arguments (Tomasello, 2003; Gentner, 2006). Evidence for a noun advantage in adult L2 acquisition has also been put forward by several studies (Gillette et al., 1999; Isurin, 2000; Snedeker and Gleitman, 2004; Gleitman et al., 2005), although there is also conflicting evidence (Ludington, 2015). Ludington (2013) further suggested that the noun advantage might be due to differences in concreteness or imageability of the target words such that more concrete words are better to memorize. This explanation, however, can be ruled out for our study as the words to be learned were evaluated to be comparable with respect to concreteness, imageability and familiarity (see section 2.2). With regard to the positive effect of pictures on verbs a possible explanation could be that the majority of verbs used in this study are abstract verbs (e.g., to think, to join, to relax, to dream) rather than manipulation verbs or locomotion verbs. de Nooijer et al. (2013) already demonstrated that (hand and arm) gestures are beneficial for memorizing manipulation verbs, but not for abstract verbs or locomotion verbs. Hence, the results of our study seem to support this prior evidence for recall of L2 abstract verbs (but not for L1 verbs). However, this also implies a limitation of our study. Future studies should explore more deeply the different effects of picture-based and gesture-based enrichments on different verb types.

Moreover, we were surprised that except for nouns gestures did not facilitate recall of L2 words. A potential explanation for this crucial finding is that imitating gestures might be more complex than observing pictures due to the additional activation of motor representations. Thus, learners' cognitive load might be higher under gesture-enrichment as compared to picture-enrichment. Under this assumption, a higher degree of cognitive load might go well with memorizing L1 words (the “easier” task), whereas it might rather impede learning L2 words due to a cognitive overload. That is, it might be that the task of gesture imitation added too much additional information to the linguistic input, so that this additional gesture and motor information interfered with the ability to memorize the L2 word and building a link with the corresponding L1 representation. This explanation could be interpreted in line with evidence provided by Kelly and Lee (2012), who found that hand gestures can disrupt word learning when learners experienced a high load due to high phonetic demands of the words to be memorized. Here, it would have been helpful to include post-test questionnaires assessing perceived cognitive load when learning words with no enrichment vs. gesture-based vs. picture-based enrichment. Moreover, future studies could include video-recordings of the learning sessions that could be analyzed regarding whether learners showed difficulties to pronounce the L2 word and simultaneously imitate the presented gesture in the gesture-condition.

Against this background and together with the above argumentation for differences in L1 vs. L2 acquisition in terms of a cognitive overload, it might be that gesture imitation, imposing a rather higher load, works better for easier-to-learn nouns, whereas picture observation imposing a lower load works better for more difficult-to-memorize (abstract) verbs. Also, some researchers suggest that attention-related problems occur in particular, when the-to-be-learned materials are of higher complexity (Sweller et al., 2011). These problems occur when learners are provided with different sources of information (as in our case L1 words, L2 words and pictures/gestures). Learners have to divide their attention over the two (or more) information sources and need to mentally integrate the information from these sources. Especially when written text and non-verbal information are presented physically separate, this results in split-attention problems (Ayres and Sweller, 2014).

There are some limitations of the present study that deserve to be mentioned. First, our measurement of individual learning style and learning preferences with the SIL inventory (Oxford, 1990) was not usable due to low degrees of internal validity. Indeed this seems to be a common problem with the learning style inventory. Researchers have put the construct's validity into question (Pashler et al., 2008) since the very limited experimental research addressing the validity of the construct failed to demonstrate a statistically significant relationship between learning style preference and instructional method for comprehension tests (e.g., Rogowsky et al., 2015). We were, therefore, not able to address individual differences among learners into our analysis and answer RQ4. However, the criticism on individual learning styles suggest that future studies should rather concentrate on other relevant variables, such as age differences or educational background. Especially with regard to the above raised question of the influence of concreteness of words (or the lack thereof) children as well as older adults might show different learning results than university students. Hence, further studies with different age groups will be necessary in order to assess how our results generalize.

Second, we did not control the detailed characteristics of our non-verbal enrichment. Although we ensured that both, gestures and pictures, were semantically congruent with the words to be learned, these might still focus on different semantic features, such as the shape of an object, or actions that can be accomplished with an entity. Moreover, as discussed above the words used in this study might not have been diverse enough as for instance verbs were predominantly abstract (as opposed to manipulating or locomotion verbs). Also, the viewpoint of a gesture might be decisive as recently observed by Kushch et al. (2016). Finally, learners were exposed to a really challenging task. They had to learn words of a language that is typologically different from their native language and were tested by free recall tests. Cued recall, multiple choice or recognition tasks certainly would have been easier and it might well be that this added to participants' cognitive load and motivation.

Despite these limitations, the results of our study suggest that the type of vocabulary learning strategy has to match with the type of linguistic material to be learned. Our findings should be replicated and refined in future research, but could have important educational implications for L2 classrooms and tutoring systems, as they show that non-verbal enrichment can foster learning, but not for all types of words alike. Therefore, we argue for a more pronounced view on the role of pictures and gestures in learning vocabularies and for more research to explore the constraints and limitations as well as the detailed mechanisms underlying the supportive function of non-verbal enrichment. Virtual learning environments incorporating pedagogical agents might play a key role in this kind of research as they enable us to manipulate even subtle aspects of behavior and situation in a re-producible way, while holding everything else stable.

## Data Availability Statement

The original contributions presented in the study are included in the article/Supplementary Material, further inquiries can be directed to the corresponding author. The pictograms used as stimulus material in this study are available upon request to the corresponding author.

## Ethics Statement

Ethical review and approval was not required for the study on human participants in accordance with the local legislation and institutional requirements. The patients/participants provided their written informed consent to participate in this study.

## Author Contributions

Both authors listed have made a substantial, direct and intellectual contribution to the work, and approved it for publication.

## Conflict of Interest

The authors declare that the research was conducted in the absence of any commercial or financial relationships that could be construed as a potential conflict of interest.
