# Supplementary figures and images for: Non-verbal Enrichment in Vocabulary Learning With a Virtual Pedagogical Agent
Source: Front Psychol. 2020 Nov 24;11:533839. doi: 10.3389/fpsyg.2020.533839 (PMC7732470; doi:10.3389/fpsyg.2020.533839)

# Supplementary Material

## 1 PLAN OF PROCEDURE

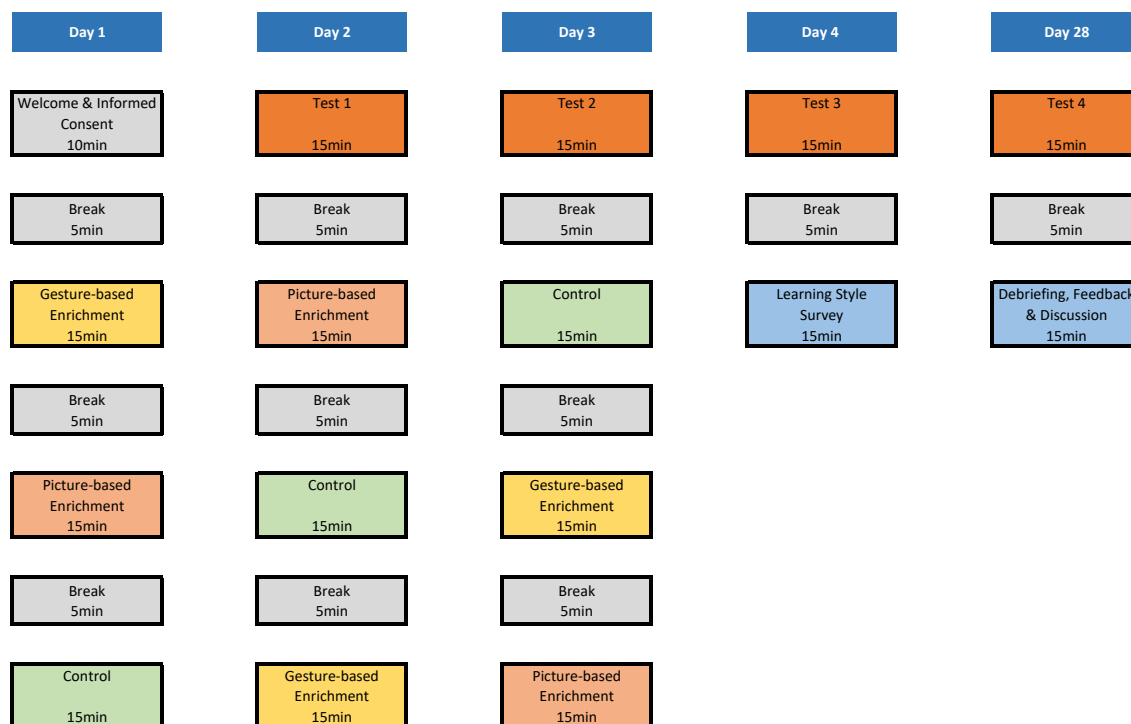

Figure S1. Plan of Procedure

Supplement: Supplementary file 4 [file Data_Sheet_4.pdf]
